# Causal relationship between ulcerative colitis and male infertility: A two-sample Mendelian randomization study

**DOI:** 10.1371/journal.pone.0303827

**Published:** 2024-05-30

**Authors:** Xia Wang, Tongyi Li, Qiu Chen

**Affiliations:** Medical Department of Endocrinology, Hospital of Chengdu University of Traditional Chinese Medicine, Chengdu, China; King Abdulaziz University Faculty of Medicine, SAUDI ARABIA

## Abstract

**Aims:**

To explore the causal relationship between ulcerative colitis (UC) and male infertility using Mendelian randomization method with single nucleotide polymorphism (SNP) as the instrumental variables.

**Methods:**

Genetic loci closely associated with UC were extracted as instrumental variables and male infertility was the outcome variable in pooled data from the gene-wide association study (GWAS),which was derived from European ethnic groups. The UC data(ebi-a-GCST003045) contained a total sample size of 27432 individuals and 110944 SNPs, and the male infertility data(finn-b-N14_MALEINFERT) contained a total sample size of 73479 individuals and 16377329 SNPs. The SNPs highly correlated with UC were screened from ebi-a-GCST003045(P<5×10^−8^ as the screening condition, the linkage disequilibrium coefficient was 0.001,and the width of the linkage disequilibrium area was 10000 kb).SNPs related to male infertility from finn-b-N14_MALEINFERT (the minimum r^2^>0.8,replacing the missing SNPs with SNPs with high linkage, and deleting SNPs without substitution sites) were extracted. MR analysis was performed using MR-Egger regression, the weighted median and the inverse-variance weighted (IVW) respectively, and the causal relationship between UC and male infertility was evaluated by OR and 95% CI, and the Egger-intercept method was used to test for horizontal multiplicity, and the sensitivity analysis was performed using "leave-one-out method". Finally, we used Bayesian Weighted Mendelian Randomization (BWMR) approach to test the results of MR study.

**Results:**

A total of 86 SNPs were included as IVs, with OR and 95% CI of 1.095(0.820~1.462)、1.059(0.899~1.248)、1.125(1.002~1.264) for MR-Egger, the weighted median and IVW results respectively, and P value of less than 0.05 for IVW, indicating that a causal relationship between UC and male infertility was causally related. The results of MR analysis combined with BWMR analysis also showed positive genetic causal relationship between UC and male infertility.MR-Egger regression showed an intercept of -2.21×10^−3^ with a standard error of 0.006 and P = 0.751, there was no horizontal pleiotropy for the IVs of exposure factors. Heterogeneity tests showed no heterogeneity and the results of the "leave-one-out" sensitivity analysis were stable.

**Conclusion:**

There is a causal association between UC and male infertility, which increases the risk of developing male infertility.

## 1.Introduction

Infertility refers to not taking contraception for more than one year without becoming pregnant in the case of regular sex [1], and the incidence of infertility in China is about 15%, of which male factors account for about 50% [2]. The diminishing quality of male reproductive fluid amidst advancing living standards has garnered burgeoning societal concern, attributable to a multifaceted array of pathogenic influences. Presently, the etiology of male infertility is thought to be intricately intertwined with reversible functional hindrances within pivotal organs like the hypothalamic-pituitary-gonadal (HPG) axis. Past exploratory inquiries into the risk determinants of male infertility were primarily fixated on endocrine and metabolic disorders, such as obesity and diabetes, acknowledged as harboring adverse implications for male reproductive fluid quality [3]. Intriguingly, ulcerative colitis (UC) has been posited as a potential catalyst for male infertility. Investigations by Friedman and Knowles [4,5] have illuminated that sexual dysfunction prevalence among males afflicted with inflammatory bowel disease (IBD) ranges from 44% to 53.9%, with erectile dysfunction (ED) emerging as the predominant manifestation of male sexual dysfunction [6,7]. Correspondingly, a questionnaire-based cross-sectional examination conducted by Zhang Jinzhi et al. [8] among 208 IBD patients, comprising 133 individuals with Crohn’s disease (CD) and 75 with UC, underscored that 44% of male IBD patients divulged experiencing ED. The dearth of systematic investigations delving into the correlation between UC and male infertility, alongside the susceptibility of observational studies to confounding variables and reverse causality [9], underscores the imperative for deploying more stringent methodologies to ascertain whether UC could potentially instigate male infertility.

In classical epidemiological investigations, the connection between exposure and outcome can be muddled by unmeasured confounders and reverse causality, casting shadows over causal deductions. Over the recent years, Mendelian randomization (MR) has emerged as a prominent tool applied to genome-wide association study (GWAS) datasets. The MR technique effectively leverages single nucleotide polymorphisms (SNPs) as instrumental variables (IVs) to delineate causal links between exposure and outcome [10]. Fundamentally, MR capitalizes on the random assortment and assortment permutations intrinsic to genetic variant transmission during gamete formation to restructure populations in a randomized manner, essentially circumventing the impact of confounding variables. Moreover, the genetic variance explicated by genetic variants (serving as IVs) takes precedence over the variance associated with the outcome, thereby obviating the quandary of reverse causation [11–13]. In this exploration, the two-sample MR approach was deployed to scrutinize the causal nexus between UC and male infertility at the genetic level, employing SNPs as instrumental variables based on GWAS summary data.

## 2.Method

### 2.1 Data sources

The investigation sourced its data from the https://gwas.Mrcieu.ac.uk/datasets website for genome-wide association studies on UC and male infertility, accessed on the 25th of June 2023. Two sets of GWAS data were extracted from European populations, irrespective of gender. The UC data (ebi-a-GCST003045) originated from GWAS statistical findings published in 2015, encompassing 27,432 individuals, comprising 6,968 cases, 20,464 controls, and 110,944 SNPs. All cases fulfilled the clinical diagnostic criteria for UC, involving comprehensive evaluations ranging from endoscopic procedures to radiological and histopathological analyses. To prevent data overlap, exposures and outcomes data were meticulously chosen from distinct databases to obviate redundancy. Genetic prognosticators of outcomes were procured from FinnGen. Conversely, the male infertility data (finn-b-N14_MALEINFERT) stemmed from GWAS statistical findings published in 2021, encompassing 73,479 individuals, with 680 cases, 72,799 controls, and 16,377,329 SNPs. Studies in the past scrutinizing fertility cognition have predominantly centered on females [14]. Clinical investigations into male infertility lag behind those concerning women, contributing to male individuals’ lack of awareness regarding the nexus between male fertility outcomes and perils related to health, such as UC. Deficiencies in understanding fertility and correlated risk factors among males could delay their pursuit of requisite treatment [15,16], culminating in a dearth of expansive genetic data studies in male infertility patient cohorts. To mitigate the repercussions of sample size imbalances in the male infertility dataset on outcomes, a Bayes-weighted Mendelian randomization (BWMR) technique for causal inference was implemented. The BWMR analysis served as a supplementary method to the two-sample MR analysis, accounting for the polygenic structure and pleiotropy of diseases or traits not encompassed by MR analysis, thereby enhancing the stability and reliability of the ultimate findings [17]. Addressing concerns regarding the potential effects of sample size imbalances on outcomes, this study conducted an exhaustive evaluation through leave-one-out sensitivity analysis and heterogeneity analysis. Horizontal pleiotropy, a plausible confounder, was identified and rectified through MR-PRESSO and MR-Egger regression intercept analyses.

According to the policy of the Ethics Committee of the Hospital of Chengdu University of Traditional Chinese Medicine, this study was exempt from ethical review due to the utilization of publicly available data and complete anonymization of patient information. Availability of data and materials.

The datasets analyzed during the current study are available in the IEU open GWAS project [https://gwas.mrcieu.ac.uk/] and FinnGen biobank [https://www.finngen.fi/en/access_results].

### 2.2 The selection of IVs

Within Mendelian randomization (MR) analysis, instrumental variables (IVs) serve as intermediary agents between exposure elements and outcomes, elucidating the causal interplay between exposure and repercussions. Typically, genetic variations function as IVs, with single nucleotide polymorphisms (SNPs) predominantly leveraged for this purpose [18]. The foundational tenets of MR analysis rest on several key assumptions. Firstly, IVs must exhibit robust associations with the exposure factors, commonly necessitating an F statistic exceeding 10 to denote a robust association [19,20]. The F statistic is computed as follows: F = r^2^ × (n–2) / (1–r^2^), where n signifies the sample size and r^2^ denotes the extent to which the instrumental variable explains the exposure. The calculation for r^2^ is: r^2^ = 2 × (1–MAF) × MAF × β^2^, with MAF representing the minimum allele frequency and β signifying the SNP effect size for an allele [21]. Secondly, IVs should not possess direct correlations with the outcome, impacting the outcome solely via the exposure, thereby evading gene pleiotropy. In this inquiry, a non-zero intercept term (P<0.05) in the MR-Egger regression model signifies the absence of gene pleiotropy [22,23]. Thirdly, IVs should remain independent of any identified or unidentified confounding variables. As the SNPs curated through the MR methodology adhere to the genetic principle of random allocation of parental alleles to progeny, their susceptibility to environmental and lifestyle influences is minuscule. Consequently, it is theoretically plausible to regard IVs as untethered from environmental variables like socioeconomic and cultural factors [24].We screened meaningful SNPs from UC’s GWAS aggregated data (P<5×10^−8^); The linkage imbalance coefficient r^2^ is set to 0.001 and the linkage imbalance region width is 10000 kb to ensure that each SNP is independent of each other and the influence of gene pleiotropy on the results is excluded [25]. The above screened male infertility-related SNPs were extracted from the GWAS summary data of UC. Set a minimum r^2^>0.8, replace the missing SNPs with SNPs that are highly interlocking with them, and delete SNPs without substitution sites [24]. Information from both datasets was pooled while excluding SNPs directly associated with male infertility (P<5×10^−8^) [26].

### 2.3 Statistical analysis

We used three regression models, MR-Egger regression, the weight median and inverse-variance weighted (IVW), using SNP as IVs to verify the causal relationship between exposure (UC) and outcome (male infertility). Among them, IVW was able to estimate the total effect of UC on male infertility [27]. Uniquely, the IVW technique facilitates the direct computation of causal effect magnitude from aggregated data without necessitating individual-level information. In instances where pleiotropy and heterogeneity are absent, the IVW outcomes, if statistically significant (P < 0.05), signal plausible results, even if other methodologies yield non-significant findings [28]. MR-Egger can be applied to both MR analysis and pleiotropy assessment, using the reciprocal of the outcome variance as a weight for fitting. The advantage of the weight median is that MR analysis can also be performed to obtain effective results if at least half of the effective IVs are analyzed [29]. BWMR, on the other hand, accommodates uncertainties intrinsic to estimations of weak effects and subtle horizontal pleiotropic influences, while adeptly identifying outliers attributed to pronounced horizontal pleiotropic effects [30]. Correlations with P < 0.05 for the raw results while P > 0.05 after adjustion were considered suggestive, whereas correlations with P < 0.05 after adjustion were considered significant [31]. Thus, we used this method to test the results by the IVW method. Cochran’s Q test was used to determine the heterogeneity of SNPs [32]; If there is heterogeneity, focus on IVW model results; The "leave-one-out method" was used for sensitivity analysis. We verified the F statistic β^2^/σ^2^ defined as mean with an F statistic of at least 10 to minimize bias in weakly genetically transmitted instruments. All analyses were performed in R software (version 4.0.4) using the TwoSampleMR package, and the test level α = 0.05.

## 3.Result

### 3.1 SNP information

In this study, UC was used as an exposure factor, and R software was used to screen SNP sites with genome-wide significance according to the screening criteria, and a total of 86 SNPs were obtained as tool variables, as shown in S1 Table. Noteworthy is the individual SNP’s F statistic value in this research, measuring at a robust 103.2027, surpassing the threshold of 10. Such a robust F statistic (>10) underscores the absence of bias stemming from feeble instrumental variables, thereby bolstering the reliability of the findings.

**Fig 1:**
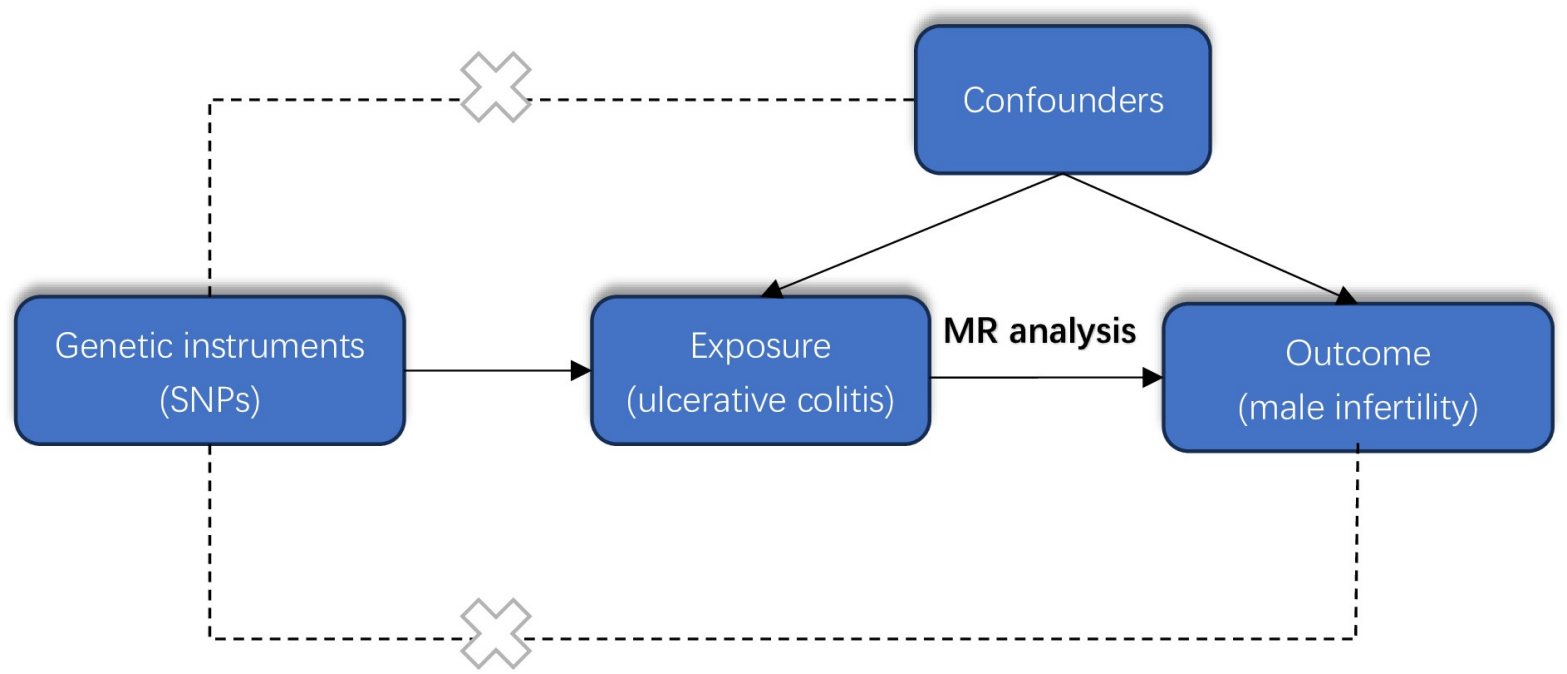
MR process: Overall design of the two-sample Mendelian randomization analysis in this study.

**Fig 2:**
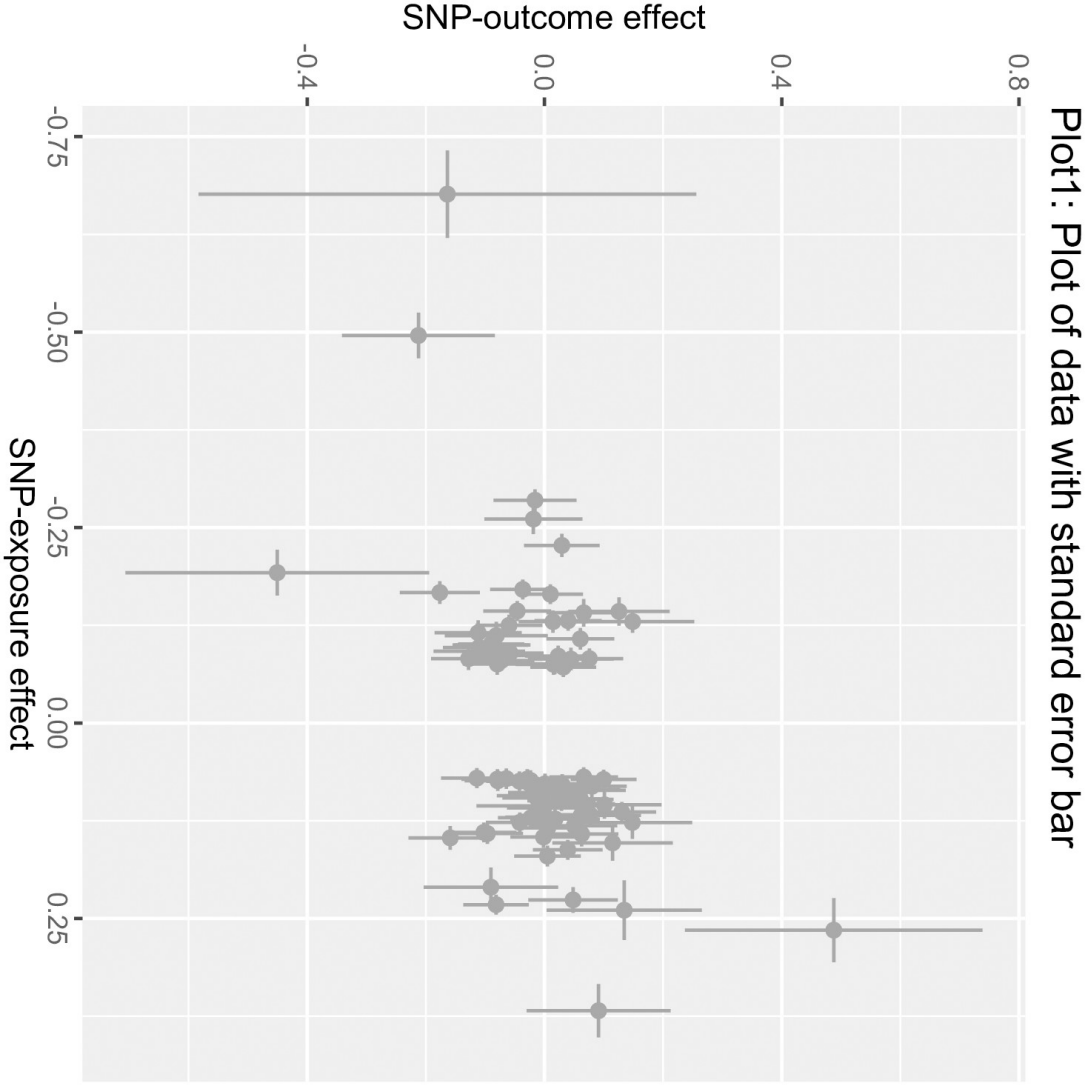
Plot of data with standard error bar: The figure showed the Bayesian Weighted Mendelian Randomization (BWMR) approach to test the data with standard error bar.

**Fig 3:**
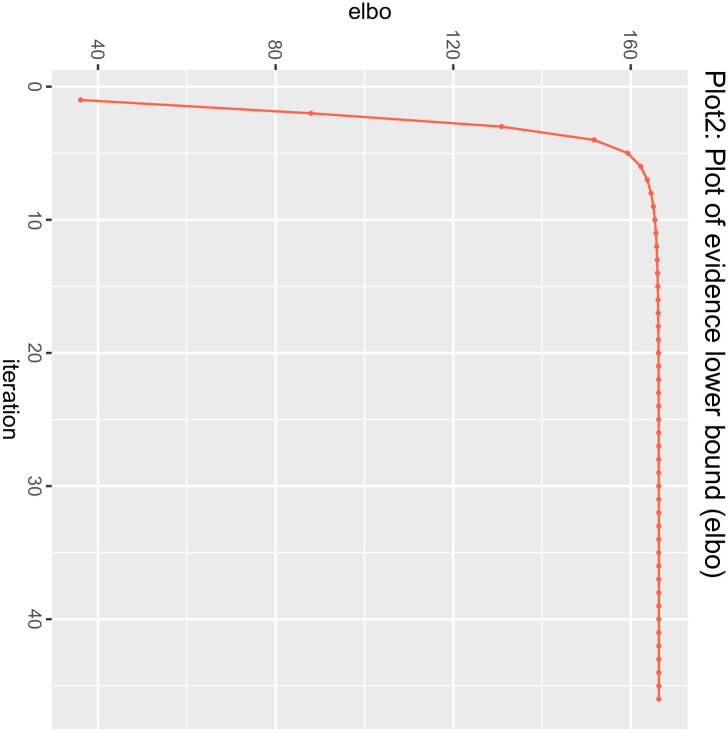
Plot of evidence lower bound: The figure showed the Bayesian Weighted Mendelian Randomization (BWMR) approach to test the evidence lower bound.

**Fig 4:**
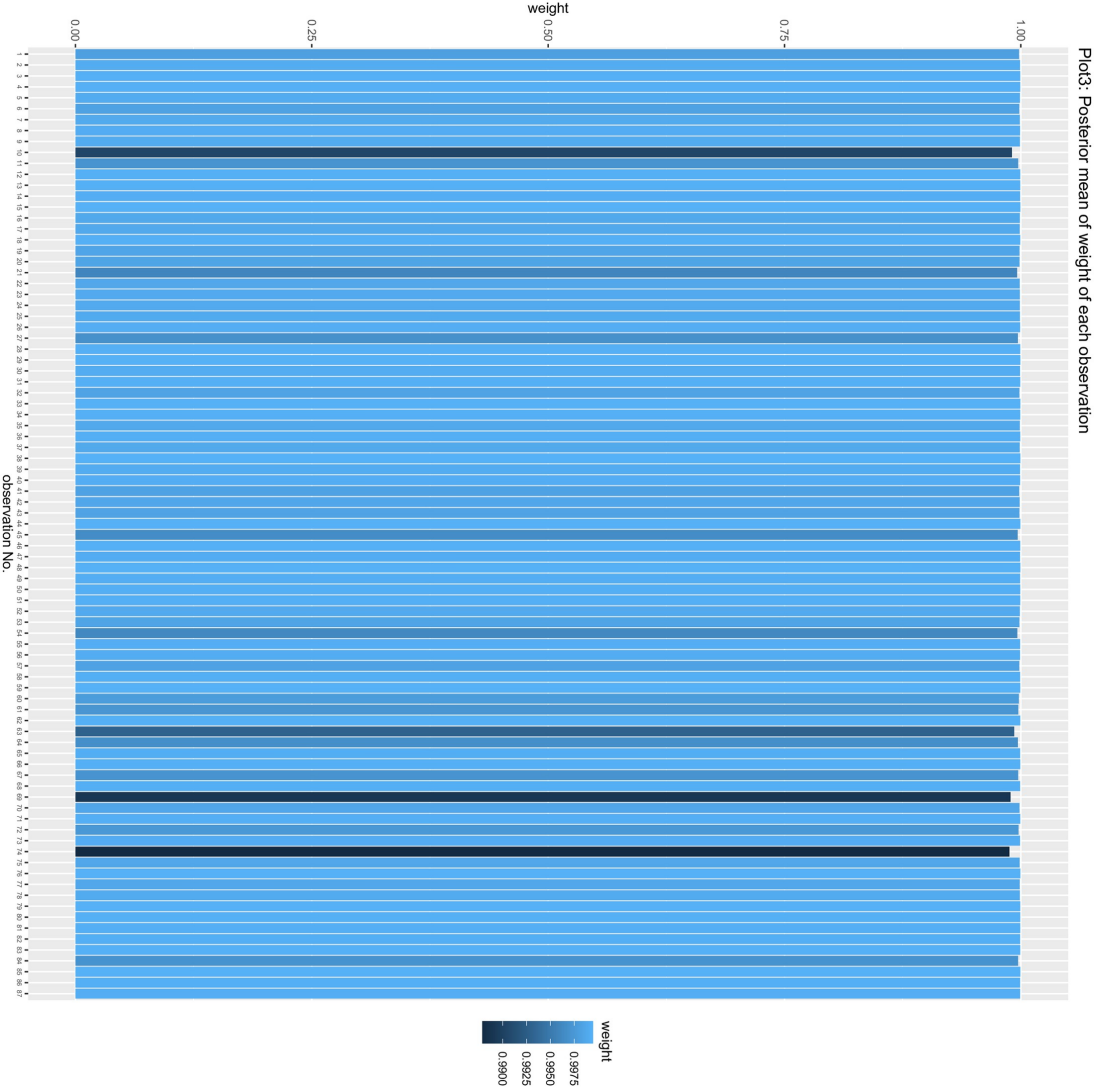
Posterior mean of weight of each observation: The Heatmap showed the Bayesian Weighted Mendelian Randomization (BWMR) approach to test the posterior mean of weight of each observation.

**Fig 5:**
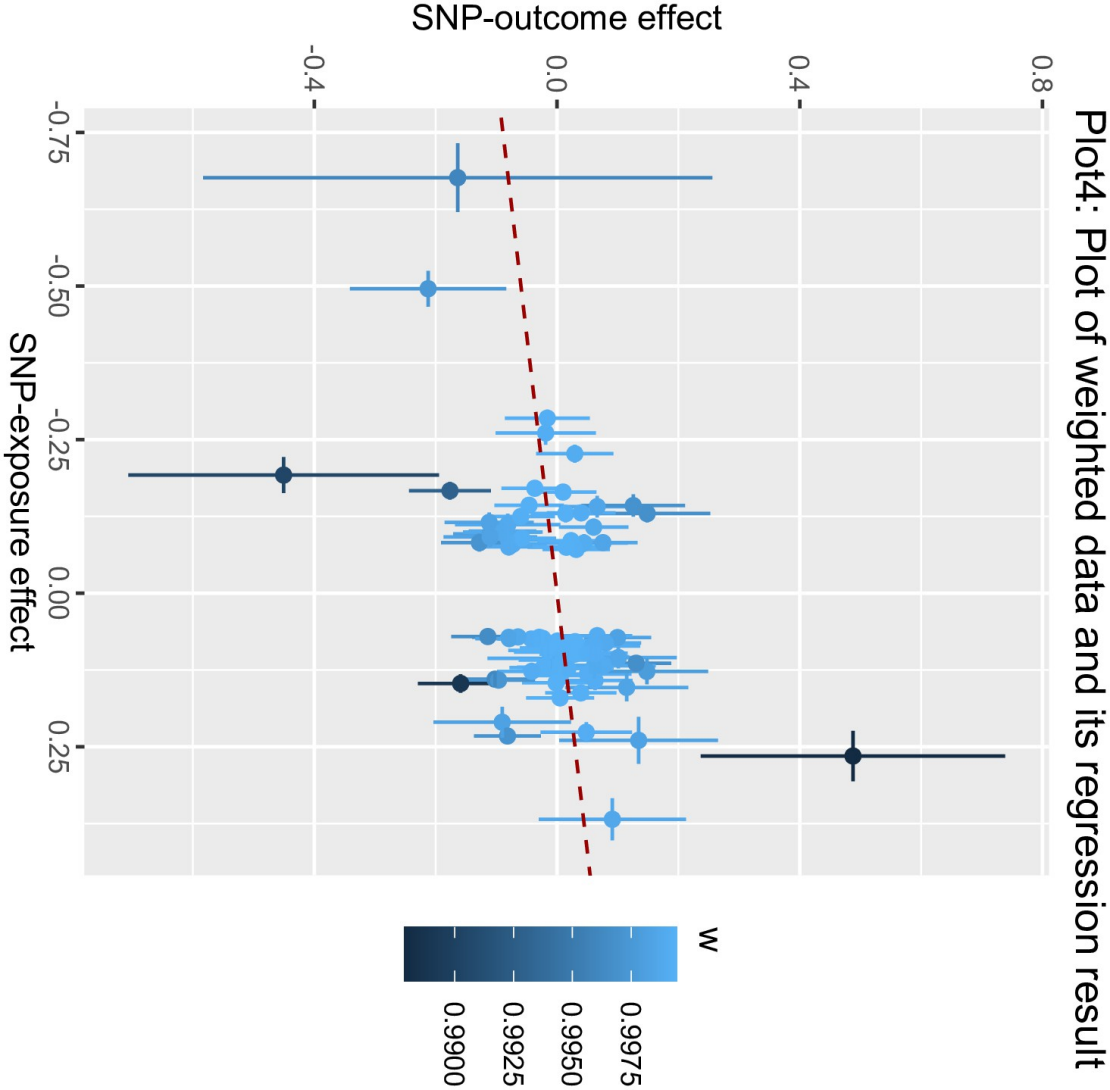
Plot of weighted data and its regression result: The plot showed the Bayesian Weighted Mendelian Randomization (BWMR) approach to test the weighted data and its regression result.

The MR process in the present study is summarized in Fig 1. The MR of UC and male infertility was analyzed using MR-Egger regression, the weight median and IVW in the TwoSample MR package, and the results are shown in Table 1, showing that the OR value and 95% CI were 1.095(0.820~1.462),1.059(0.899~1.248) and 1.125 (1.002~1.264), respectively. From the results, it can be seen that MR-Egger regression, the weight median and IVW results all show that people with UC increase the risk of male infertility, but the P values of the three test methods are 0.538, 0.487, 0.045, the results of IVW are less than 0.05, the difference is statistically significant, the results of MR-Egger and the weight median are greater than 0.05, and the β values of the three analysis methods are consistent in the direction, subject to the IVW results, so the angle of MR analysis can be obtained. Upon subjecting the data to scrutiny via the BWMR method, a P-value of 0.04628 emerged (as presented in Figs 2–5), indicating significance (P < 0.05 post-adjustment). This outcome underscores a heightened risk of male infertility among individuals with UC, thereby substantiating a causal nexus between UC and male infertility.

**Table 1:** MR results of ulcerative colitis in male infertility.

method	β	SE	OR (95%CI)	P 值
**MR Egger**	**0.091124**	**0.147503**	**1.095(0.820~1.462)**	**0.538396**
**WME**	**0.057979**	**0.083571**	**1.059(0.899~1.248)**	**0.487827**
**IVW**	**0.118542**	**0.059326**	**1.125(1.002~1.264)**	**0.045703**

Table 1. MR results of ulcerative colitis in male infertility: Associations between genetically predicted ulcerative colitis and risk of male infertility.

### 3.2 Sensitivity analysis

**Fig 6:**
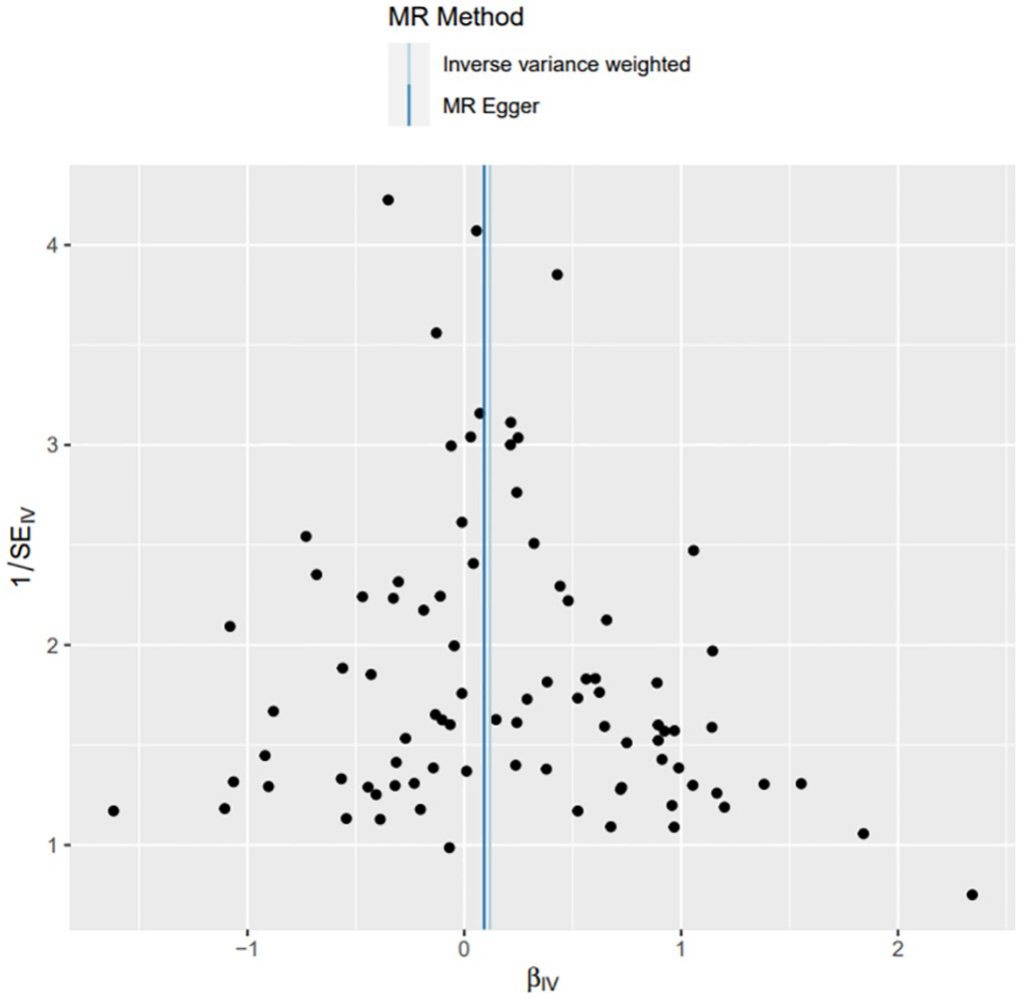
Funnel chart of MR method analysis: Funnel chart indicates that there is no heterogeneity in the results.

**Fig 7:**
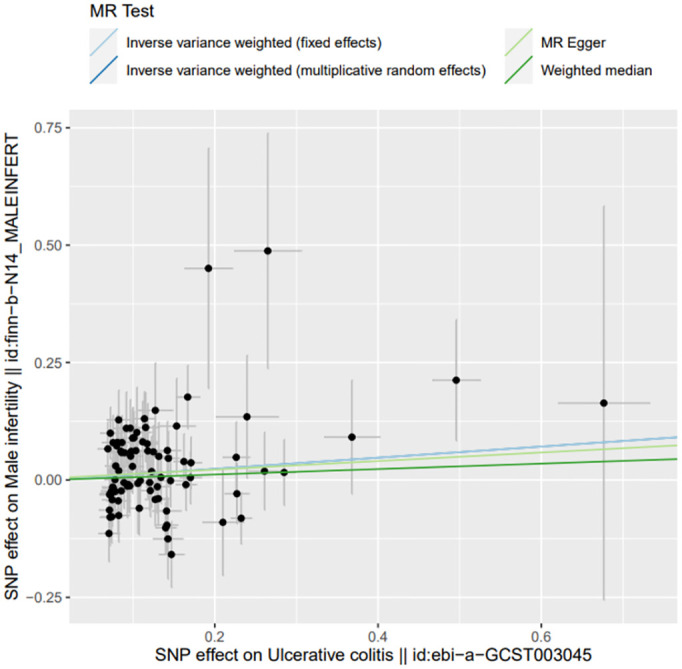
Scatter plot of heterogeneity test results: Scatter plot depicting the distribution of individual ratio estimates of ulcerative colitis with male infertility as the outcome.

Throughout this investigation, adherence to stringent screening criteria for instrumental variables (IVs) was paramount, coupled with the inclusion of a homogeneous population cohort, thereby mitigating the likelihood of false negative outcomes. Notably, the Q statistics and QP values associated with MR-Egger and IVW methodologies appeared as 57.345 (0.388) and 57.451 (0.421) respectively, surpassing the 0.05 threshold, thus signifying homogeneity within the data. The outcomes were aptly visualized, with presentations of funnel and scatterplots articulated in Figs 6 and 7.

The MR-Egger regression unveiled an intercept of -2.21×10^-3, accompanied by a standard error of 0.006 and a p-value of 0.751, signifying the absence of horizontal pleiotropy concerning the instrumental variables associated with the exposure factors. Notably, heterogeneity assessments revealed no discernible heterogeneity, while the outcomes from the "leave-one-out" sensitivity analysis remained steadfast and reliable.

The IVW method was visualized by the sensitivity analysis "Leave-one-out" method, and after removing a single SNP in turn, the IVW effect values of the remaining SNPs did not fluctuate greatly, they were close to the red dot position in the figure, and the P value results were all greater than 0.05, indicating that no matter any SNP removed, it would not have a great impact on the results, indicating that the MR results of this study were robust. (S1 and S2 Figs).

## 4.Discussion

Due to a myriad of factors such as social and cultural influences, physicians frequently encounter challenges in broaching discussions with patients regarding matters pertaining to sexual functionality. Regrettably, the potential risk of sexual dysfunction linked to Inflammatory Bowel Disease, specifically Ulcerative Colitis, often remains inadequately acknowledged during the diagnostic and therapeutic processes [33,34]. Consequently, the correlation between Ulcerative Colitis and male infertility tends to be disregarded, emerging as a pivotal element impacting the physical and psychological well-being of afflicted individuals. Remarkably, Zhang Jinzhi et al [8] observed that patients exhibiting moderate to severe disease activity in Ulcerative Colitis display a heightened susceptibility to developing sexual dysfunction compared to those in disease remission, with the severity of such dysfunction escalating in tandem with disease activity. Furthermore, research indicates an absence of significant associations between Ulcerative Colitis-related medications, surgical history, and the occurrence of sexual dysfunction.

In this study, the causal relationship between UC and male infertility in the organism was studied and investigated by MR analysis combined with BWMR analysis for the first time. This study uses a genetic approach to provide IVW results based on the causal relationship between UC and anthropometric characteristics associated with male infertility, showing that each patient with UC has a fold increased risk of male infertility compared to the normal population. While the derived P-values oscillate between faint significance (0.045) and insignificance (0.538 and 0.487), the Leave-one-out examination indicated that individual SNPs failed to significantly skew the MR results. Therefore, the primary outcome should be considered an IVW result since there was no significant heterogeneity or unbalanced pleiotropy regarding the risk of diabetic neuropathy. And the IVW method result, which yielded a P-value of 0.045, indicating a statistically significant relationship between UC and male infertility. Furthermore, the unanimity of effect direction observed across all methodologies (MR-Egger, weighted median, IVW) buttresses the putative validity of our observations, notwithstanding the variability in P-value salience. Consequently, our revelations bear pivotal clinical relevance across the spectrum of UC and male infertility etiology, diagnosis, therapy, and prophylaxis. Studies in recent years [35] have found that endothelial dysfunction may lead to ED in IBD patients represented by UC. UC can cause microvascular endothelial dysfunction, characterized by loss of nitric oxide (NO)-dependent dilation, resulting in decreased microvascular perfusion, poor wound healing, and persistent chronic inflammation, which in turn causes the development of ED. Simultaneously, UC, as a chronic inflammatory colonic disorder exemplified by enduring mucosal devastation and regeneration, frequently coincides with derangements in intestinal microbiota. Studies have shown that the gut microbiota can metabolize nutrients in the gut and can also regulate intestinal metabolites to affect the blood metabolome [36]. Disorders of intestinal microbiota metabolism in UC patients will further affect the homeostasis of glucose metabolism in the body, and glucose metabolism disorders can affect the sugar utilization process of hormone-secreting cells such as Leydig cells, damage sex hormone synthesis and lead to insufficient energy sources required for sperm activity, further causing male sexual dysfunction [37].

Moreover, ZHANG et al. [38] elucidated that within a metabolic syndrome model induced by excessive dietary intake in sheep, disturbed spermatogenesis was intricately intertwined with aberrant vitamin A metabolism. Intestinal dysbiosis, notably characterized by a decline in the relative abundance of specific taxa within the rumen bulb cohort, correlates with a notable reduction in bile acid levels, subsequently impinging on vitamin A absorption and culminating in compromised spermatogenesis. Several studies have confirmed that probiotics have both therapeutic effects on UC and improve sperm quality. As an example, the results of a study evaluating the effects of probiotic milk (containing Bifidobacteria and Lactobacillus acidophilus) in patients with UC during remission in UC showed significant improvements in clinical activity index and histologic scores in the probiotic group compared with placebo [39]. Studies [40] found that treatment of DSS-induced UC mice with Saccharomyces boulardii could reduce their inflammatory response and intestinal mucosal damage, and the mechanism may be related to increasing pyruvate kinase M2. In terms of improving sperm quality, a dietary supplement with a probiotic (Lactobacillus reuteri) increased spermatogenic tubular cross-section, spermatogenesis, and the number of Leydig cells in aging mice, thereby helping aging mice maintain their testicular size and rejuvenation of testosterone levels [41]. Treatment with probiotics (lactobacillus and bacillus) in rats on a high-fat diet prevented oxidative stress of sperm and associated degradation of sperm quality [42]. The above studies have shown that for male patients with a clear diagnosis of UC, probiotic therapy should be used early to prevent the exacerbation of dysfunction, and it has a good effect on the remission and improvement of both.

Different from the previous observational studies are susceptible to genetic, immune and other interference factors, RCT experiments are difficult to carry out a large number of disadvantages, this MR analysis study uses public GWAS data, the results are open and transparent, the selected data populations are European race, to avoid bias results caused by racial factors. Notably, comprehensive heterogeneity assessments were conducted, with IVW and MR-Egger QP values exceeding 0.05, signaling the absence of heterogeneity. Likewise, pleiotropy testing yielded P-values surpassing 0.05, affirming the absence of pleiotropic influences. Furthermore, robustness was ensured through stable and dependable results from the sensitivity analysis employing the "leave-one-out" technique. The amalgamation of MR analysis with BWMR scrutiny unveiled post-adjustment statistical significance with P-values falling below 0.05, underscoring the substantial implications of the findings.

## 5.Limitations

The limitations of this study primarily center on the following aspects: Firstly, the conclusions drawn from this investigation are specifically based on individuals of European descent, necessitating further research and validation to generalize findings to other populations. At present, there are few GWAS data in Asian and Chinese clusters, and data from other databases are difficult to obtain and collate, so the results of this study need to be verified in Chinese clusters in combination with clinical and randomized controlled trials. Secondly, the significant imbalance of sample sizes in the male infertility dataset (680 cases and 72,799 controls) may promote concerns about the reliability of the GWAS summary data. To mitigate this concern, stringent criteria were applied in instrumental variable selection, effectively attenuating the impact of sample size imbalances on study outcomes. Existing studies endorse the capacity of BWMR to enhance result stability, thus, our validation of MR analysis results using BWMR successfully addresses sample size discrepancies. Notably, findings from Fan et al.’s investigation [43] utilizing male infertility data from the FinnGen database also underscore minimal impact from sample size disparities, bolstering the reliability of our methods through a balanced approach to horizontal pleiotropy. Third, MR analysis can only explore causality, but cannot study specific biological mechanisms. Larger-scale studies with expanded sample sizes may offer clarity on observed associations and yield more compelling statistical significance. Delving deeper into the biological underpinnings of the UC-male infertility nexus could provide pivotal evidence supporting causal relationships beyond statistical outputs. Moreover, a number of P-values falling short of conventional statistical thresholds may stem from limitations in sample sizes or a paucity of effective SNP markers. Furthermore, the absence of stratified analysis results such as gender, age, and disease duration in the extracted GWAS data impedes a nuanced exploration of specific information, potentially impacting the interpretation of P-values. To tackle these challenges, we conducted sensitivity analyses and leveraged non-overlapping summary-level data to mitigate bias, thereby fortifying the overall robustness of our conclusions.

Moreover, the absence of statistically significant heterogeneity between UC and male infertility was indicated by results from IVW and MR-Egger tests for heterogeneity assessment. Furthermore, the application of BWMR—a statistical method for causal inference based on GWAS summary statistics—exhibits proficiency in addressing weak effect uncertainties and horizontal pleiotropic effects, along with the capacity to detect outliers amidst limited instances of significant horizontal pleiotropy. The combined analyses of MR and BWMR underscore a positive genetic causal link between UC and male infertility. To buttress the veracity of our findings, exhaustive sensitivity analyses have been conducted, further enhancing the credibility of our results. Additionally, where viable, we have considered meta-analysis approaches to bolster evidence through aggregated data examination.

## 6.Conclusion

In essence, this research employed UC as an exposure variable, identified SNPs significantly associated with the condition as instrumental variables, and utilized MR-Egger regression, weight median, and IVW methods for analysis. Sensitivity analyses revealed the absence of pleiotropy and heterogeneity. Furthermore, we leveraged the BWMR approach for causal inference. The amalgamated findings of MR and BWMR analyses unveiled a favorable genetic causal connection between UC and male infertility. The outcomes indicated that individuals with UC faced elevated risks of male infertility, implying the importance for UC patients to consider sexual function attentively throughout their treatment course.

## Supporting information

S1 Fig Leave-one-out sensitivity analysis. Leave-one-out sensitivity analysis for ulcerative colitis on male infertility. The dark dots in the visualization represent effect measures derived through IVW-MR analysis, with the exclusion of specific SNPs. Red lines denote the pooled analysis, incorporating all SNPs through the IVW-MR method, and are plotted for the purpose of comparison.
(TIF)

S2 Fig Forest plot. Forest plot depicting the causal effect of each single SNP on the risk of male infertility.
(TIF)

S1 Table Basic information table of SNPs associated with ulcerative coliti: Ulcerative colitis SNPs used to construct the instrument variable in Europeans. (CSV)

## References

[1] N P S, Mark S, Barbara C, et al. Diagnosis and Treatment of Infertility in Men: AUA/ASRM Guideline Part I.[J]. The Journal of urology,2021,205(1). 10.1097/JU.0000000000001521 33295257

[2] Recent advances in understanding and managing male infertility[J]. F1000Research,2019,8. doi: 10.12688/f1000research.17076.1 31143441 PMC6524745

[3] Lingbo LUO, Yang MU, Jing YANG. Endocrine and metabolic diseases and male infertility[J].J ournal of Reproductive Medicine,2023,32(01):133–137.

[4] Friedman S. Sexual Dysfunction in Inflammatory Bowel Disease: “Don’t Ask, Don’t Tell” Doesn’t Work. Inflamm Bowel Dis (2015) 21:948–50. doi: 10.1097/MIB.0000000000000259 25789924

[5] Knowles SR, Gass C, Macrae F. Illness Perceptions in IBD Influence Psychological Status, Sexual Health and Satisfaction, Body Image and Relational Functioning: A Preliminary Exploration Using Structural Equation Modeling. J Crohns Colitis (2013) 7(9):e344–50. doi: 10.1016/j.crohns.2013.01.018 23453888

[6] Mahmood S, Nusrat S, Crosby A, Zhao YD, Ali T. Assessment Of Sexual Function Among Inflammatory Bowel Disease Patients. Am J Gastroenterol (2015) 110:601–3. doi: 10.1038/ajg.2015.53 25853205

[7] Mantzouranis G, Fafliora E, Glanztounis G, Christodoulou DK, Katsanos KH. Inflammatory Bowel Disease and Sexual Function in Male and Female Patients: An Update on Evidence in the Past Ten Years. J Crohns Colitis (2015) 9:1160–8. doi: 10.1093/ecco-jcc/jjv140 26254470

[8] Jinzhi Z, Jiao N, Min Z, et al. Prevalence and Associated Factors of Sexual Dysfunction in Patients With Inflammatory Bowel Disease[J]. Frontiers in Endocrinology,2022,13. 10.3389/fendo.2022.881485 PMC9094619 35573991

[9] Davey G S, Shah E. ’Mendelian randomization’: can genetic epidemiology contribute to understanding environmental determinants of disease?[J]. International journal of epidemiology,2003,32(1). 10.1093/ije/dyg070 12689998

[10] Andreas Ziegler, Friedrich Pahlke, König Inke R. Comments on ’Mendelian randomization: using genes as instruments for making causal inferences in epidemiology’ by Lawlor Debbie A., Harbord R. M., Sterne J. A., Timpson N. and Smith G. Davey, Statistics in Medicine, doi: 10.1002/sim.3034[J]. Statistics in medicine,2008,27(15). 17886233

[11] Lingbo LUO, Yang MU, Jing YANG. Endocrine and metabolic diseases and male infertility[J].Journal of Reproductive Medicine,2023,32(01):133–137.

[12] LUO Yangyang. Mendelian randomization analysis of two samples of alcohol consumption and causal association with common mental illness[D].Jining Medical College,2022..

[13] WANG Shihao, FU Sai, ZHAO Yu, et al. Two Mendelian randomized studies on the causal relationship between renal function and osteoporosis[J]. Modern Prev Medicine,2022,49(09):1537–1542+1589.

[14] Bretherick K. L., Fairbrother N., Avila L., Harbord S. H., & Robinson W. P. (2010). Fertility and aging: Do reproductive-aged Canadian women know what they need to know? Fertility and Sterility, 93(7), 2162–2168. doi: 10.1016/j.fertnstert.2009.01.064 19296943

[15] Harrison C., Greves G., Barnard E., Davies A., McEleny K., Gordon U., et al. (2023). The effect of an educational animation on knowledge of testicular health and fertility of adolescents. Human Reproduction, 38(12), 2470–2477. doi: 10.1093/humrep/dead195 37805989 PMC10694399

[16] Krishnan S, Daly MP, Kipping R, et al. A systematic review of interventions to improve male knowledge of fertility and fertility-related risk factors. HUM FERTIL. 2024; 27 (1): 2328066. doi: 10.1080/14647273.2024.2328066 38497245

[17] Zhao J, Ming J, Hu X et al. Bayesian weighted Mendelian randomization for causal inference based on summary statistics[J]. Bioinformatics, 2020, 36(5):1501–1508. doi: 10.1093/bioinformatics/btz749 . 31593215

[18] Wenwen Y, Yanjiang Y, Li H, et al. Dietary factors and risk for asthma: A Mendelian randomization analysis [J]. Frontiers in Immunology,2023,14. 10.3389/fimmu.2023.1126457 PMC9992976 36911739

[19] QIN Youyi. Research and application of instrumental variable method based on multi-level model[D].Second Military Medical University,2015

[20] Boggs Jennifer M, Beck Arne, Ritzwoller Debra P, Battaglia Catherine, Anderson Heather D, Lindrooth Richard C. A Quasi-Experimental Analysis of Lethal Means Assessment and Risk for Subsequent Suicide Attempts and Deaths.[J].Journal of general internal medicine,2020,35(6). doi: 10.1007/s11606-020-05641-4 32040838 PMC7280370

[21] Jiahao Ding, Mengqi Zhang, Mingxia Hao, et al. Mendelian randomized study on the causal relationship between schizophrenia and suicide or intentional self-harm[J].Chinese Journal of Psychiatry,2023,56(01):32–39.

[22] L B P, Habibul A, J T V. Power and instrument strength requirements for Mendelian randomization studies using multiple genetic variants.[J].International journal of epidemiology,2011,40(3). 10.1093/ije/dyq151 PMC3147064 20813862

[23] Stephen Burgess, Thompson Simon G. Interpreting findings from Mendelian randomization using the MR-Egger method.[J]. European journal of epidemiology,2017,32(5). 10.1007/s10654-017-0255-x PMC5506233 28527048

[24] Xuan YANG, Yanzhi LI, Wei MA, Chongqi JIA. Causal relationship between lung function randomized by Mendel and mortality risk of novel coronavirus pneumonia based on two samples[J]. Journal of Shandong University(Health Sciences),2021,59(07):104–111.

[25] Gibran Hemani, Jie Zheng, Benjamin Elsworth, Wade Kaitlin H Haberland Valeriia, Denis Baird, et al The MR-Base platform supports systematic causal inference across the human phenome.[J]. eLife,2018,7. 10.7554/eLife.34408 PMC5976434 29846171

[26] Cui Z, Tian Y. Using genetic variants to evaluate the causal effect of serum vitamin D concentration on COVID-19 susceptibility, severity and hospitalization traits: a Mendelian randomization study. J Transl Med. 2021; 19 (1): 300. doi: 10.1186/s12967-021-02973-5 34246301 PMC8271325

[27] Chen X, Kong J, Diao X, et al. Depression and prostate cancer risk: A Mendelian randomization study. Cancer Med. 2020; 9 (23): 9160–9167. doi: 10.1002/cam4.3493 33027558 PMC7724297

[28] Pires F H, Martin N D, Gibran H, et al. Two-sample Mendelian randomization: avoiding the downsides of a powerful, widely applicable but potentially fallible technique.[J]. International journal of epidemiology,2016,45(6). 10.1093/ije/dyx028 PMC5722032 28338968

[29] Yuping SHU, Danqi YU, Yue RONG, et al. Causal relationship between ulcerative colitis and bronchiectasis based on Mendelian randomization[J].Chinese Journal of General Practice,2023,21(06):924–926+1043.

[30] Zhao Jia, Ming Jingsi, Hu Xianghong, Chen Gang, Liu Jin, Yang Can, Bayesian weighted Mendelian randomization for causal inference based on summary statistics, Bioinformatics, Volume 36, Issue 5, March 2020, Pages 1501–1508, doi: 10.1093/bioinformatics/btz749 31593215

[31] Li Y, Lai S, Kan X. Causal relationship between immune cells and telomere length: mendelian randomization analysis. BMC Immunol. 2024; 25 (1): 19. doi: 10.1186/s12865-024-00610-6 38459464 PMC10924351

[32] Jack B, Wesley S, Fabiola M G D, et al. Improving the visualization, interpretation and analysis of two-sample summary data Mendelian randomization via the Radial plot and Radial regression.[J]. International journal of epidemiology,2018,47(4). 10.1093/ije/dyy101 PMC6124632 29961852

[33] Rivière P, Zallot C, Desobry P, Sabaté JM, Vergniol J, Zerbib F, et al. Frequency of and Factors Associated With Sexual Dysfunction in Patients With Inflammatory Bowel Disease. J Crohns Colitis (2017) 11:1347–52. doi: 10.1093/ecco-jcc/jjx100 28981625

[34] O’Toole A, de Silva PS, Marc LG, Ulysse CA, Testa MA, Ting A, et al. Sexual Dysfunction in Men With Inflammatory Bowel Disease: A New IBD-Specific Scale. Inflamm Bowel Dis (2018) 24:310–6. doi: 10.1093/ibd/izx053 29361102 PMC6014620

[35] Hatoum OA, Binion DG, Otterson MF, Gutterman DD, et al. Acquired Microvascular Dysfunction in Inflammatory Bowel Disease: Loss of Nitric Oxide-Mediated Vasodilation. Gastroenterology (2003) 125(1):58–69. doi: 10.1016/s0016-5085(03)00699-1 12851871

[36] Ruixin L, Jie H, Xiaoqiang X, et al. Gut microbiome and serum metabolome alterations in obesity and after weight-loss intervention.[J]. Nature medicine,2017,23(7). 10.1038/nm.4358 28628112

[37] GOU Jiang, ZHAO Jun, ZHAO Jing, et al. Meta-analysis of the effect of male body mass index on sperm quality and sex hormone levels[J].Modern Journal of Urology,2019,24(06):461–466.

[38] Teng Z, Peng S, Qi G, et al. Disrupted spermatogenesis in a metabolic syndrome model: the role of vitamin A metabolism in the gut-testis axis.[J].Gut,2021. 10.1136/gutjnl-2020-323347 PMC8666830 33504491

[39] Jayshree M, Madyson S, Longxiang K, et al. Inflammatory Bowel Disease Therapeutics: A Focus on Probiotic Engineering.[J]. Mediators of inflammation,2022,2022. 10.1155/2022/9621668 PMC8786545 35082553

[40] BU Nan, FAN Yanqiu, ZHAO Chunhong. Study on the role and mechanism of Saccharomyces bralardii in the treatment of ulcerative colitis based on gut-specific pyruvate kinase M2 knockout mice[J].Journal of Practical Medicine,2020,36(12):1628–1633..

[41] Poutahidis T, Springer A, Levkovich T, et al. Probiotic microbes sustain youthful serum testosterone levels and testicular size in aging mice.[J]. PLoS ONE,2017,9(1). 10.1371/journal.pone.0084877 PMC3879365 24392159

[42] CHEN XL GONG LZ XU JX. Antioxidative activity and protective effect of probiotics against high-fat diet-induced sperm damage in rats[J]. Animal,2013,7(2):287–292. doi: 10.1017/S1751731112001528 23031185

[43] Fan C, Zhang J, Qiu D. Causal relationship between genetically predicted type 2 diabetes mellitus and male infertility. Front Endocrinol (Lausanne). 2024; 15 1357279. doi: 10.3389/fendo.2024.1357279 38529400 PMC10961381

