# Supplementary figures and images for: Causal relationship between ulcerative colitis and male infertility: A two-sample Mendelian randomization study
Source: PLoS One. 2024 May 30;19(5):e0303827. doi: 10.1371/journal.pone.0303827 (PMC11139326; doi:10.1371/journal.pone.0303827)

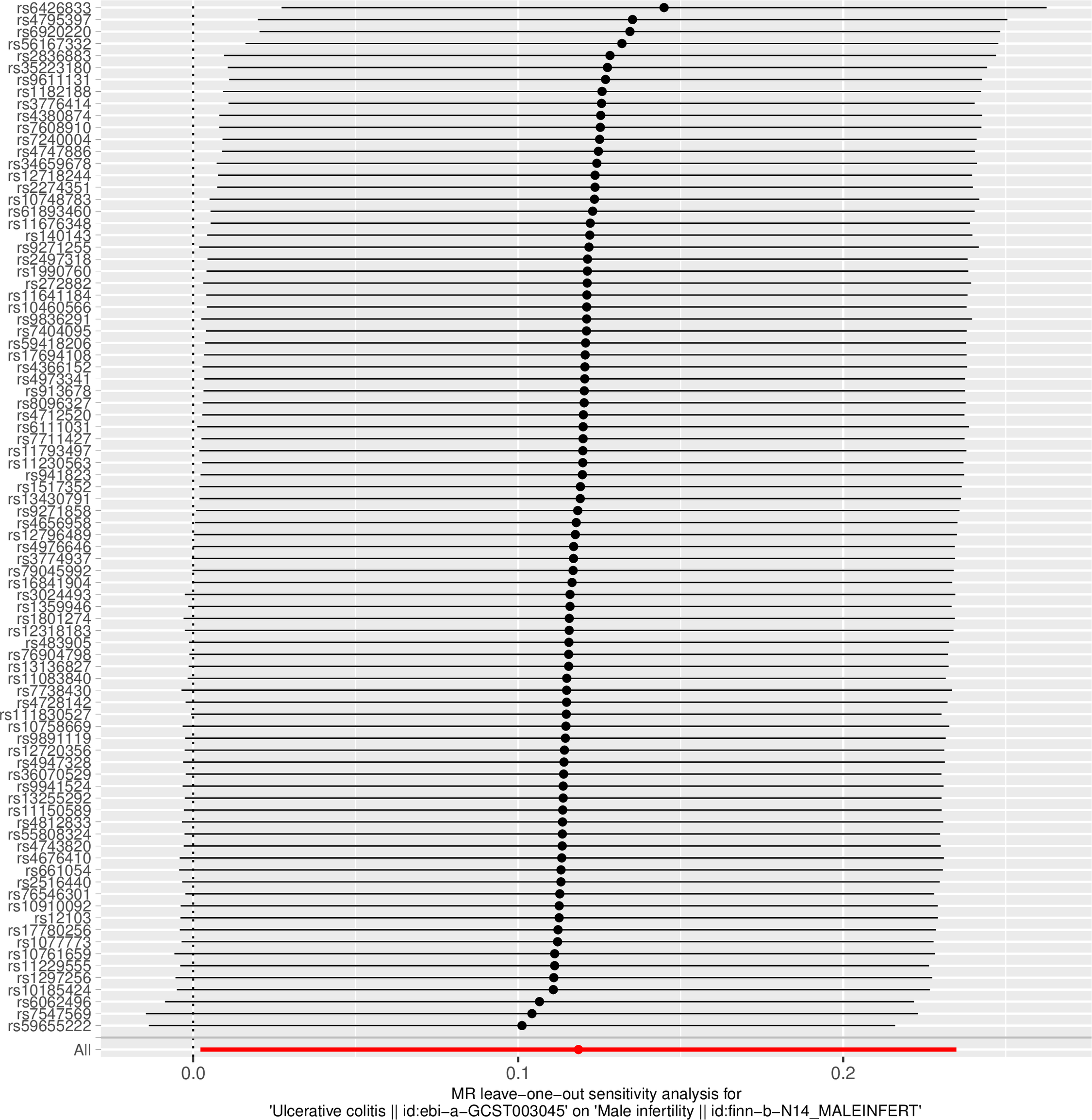

Supplement: S1 Fig — Leave-one-out sensitivity analysis for ulcerative colitis on male infertility. The dark dots in the visualization represent effect measures derived through IVW-MR analysis, with the exclusion of specific SNPs. Red lines denote the pooled analysis, incorporating all SNPs through the IVW-MR method, and are plotted for the purpose of comparison. (TIF) [file pone.0303827.s001.tif]

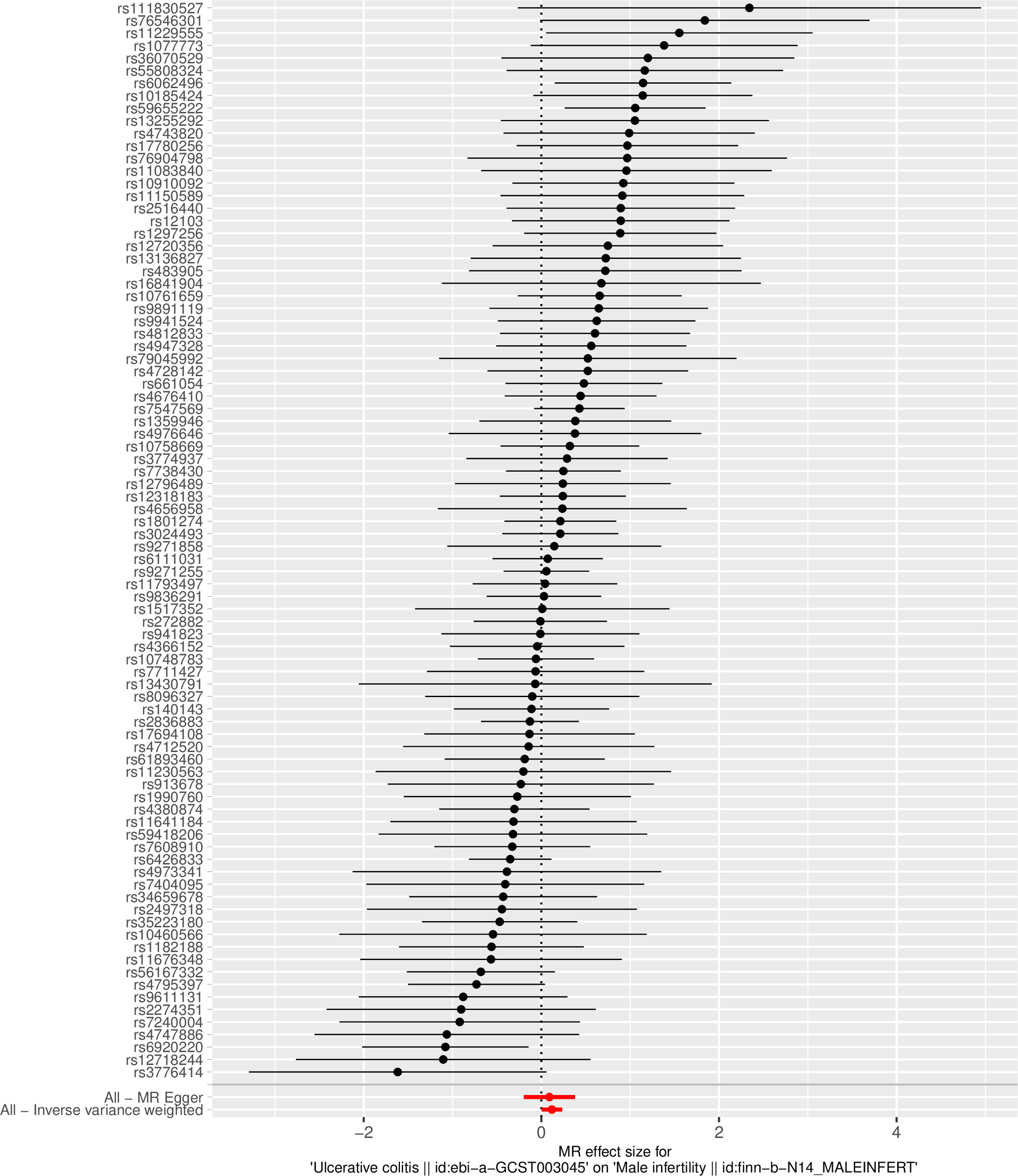

Supplement: S2 Fig — Forest plot depicting the causal effect of each single SNP on the risk of male infertility. (TIF) [file pone.0303827.s002.tif]
